# Evaluating safety risks of whole-body cryotherapy/cryostimulation (WBC): a scoping review from an international consortium

**DOI:** 10.1186/s40001-023-01385-z

**Published:** 2023-09-28

**Authors:** Fabien D. Legrand, Benoît Dugué, Joe Costello, Chris Bleakley, Elzbieta Miller, James R. Broatch, Guillaume Polidori, Anna Lubkowska, Julien Louis, Giovanni Lombardi, François Bieuzen, Paolo Capodaglio

**Affiliations:** 1 Laboratoire C2S, EA 6291, Université de Reims Champagne Ardennes, 51100 Reims, France; 2 Laboratoire Mobilité Vieillissement, Exercice (MOVE), UR 20296, Faculté des Sciences du Sport, Université de Poitiers, 86000 Poitiers, France; 3 Extreme Environments Laboratory, School of Sport, Health and Exercise Science, University of Portsmouth, Portsmouth, England, UK; 4 Faculty of Life and Health Sciences, Ulster University, York St, Belfast, BT15 1ED UK; 5 Department of Neurological Rehabilitation, Medical University of Lodz, Milionowa 14, Lodz, Poland; 6 Institute for Health and Sport (IHES), Victoria University, Melbourne, Australia; 7 MATIM, Université de Reims Champagne Ardennes, 51100 Reims, France; 8 Department of Functional Diagnostics and Physical Medicine, Pomeranian Medical University in Szczecin, Żołnierska 54, 71-210 Szczecin, Poland; 9 Research Institute for Sport and Exercise Sciences (RISES), Liverpool John Moores University, Liverpool, L3 3AF UK; 10 Laboratory of Experimental Biochemistry, IRCCS Istituto Ortopedico Galeazzi, 20157 Milan, Italy; 11 Service des Sciences du Sport, Institut National du Sport du Québec, Montréal, QC Canada; 12 Laboratorio di Ricerca in Biomeccanica, Riabilitazione ed Ergonomia, Università di Torino, Torino, Italy

**Keywords:** Whole-Body Cryotherapy/Cryostimulation (WBC), Adverse events, Scoping review, International consortium

## Abstract

Over the two last decades, whole-body cryotherapy/cryostimulation (WBC) has emerged as an exciting non-pharmacological treatment influencing inflammatory events at a cellular and physiological level, which can result in improved sleep quality, faster neuromuscular recovery after high-intensity exercise, and chronic pain relief for patients suffering different types of diseases (fibromyalgia, rheumatism, arthritis). Some evidence even suggests that WBC has benefits on mental health (depression, anxiety disorders) and cognitive functions in both adults and older adults, due to increased circulating BDNF levels. Recently, some safety concerns have been expressed by influential public health authorities (e.g., FDA, INSERM) based on reports from patients who developed adverse events upon or following WBC treatment. However, part of the data used to support these claims involved individuals whose entire body (except head) was exposed to extreme cold vaporized liquid nitrogen while standing in a narrow bathtub. Such a procedure is known as partial-body cryotherapy (PBC), and is often erroneously mistaken to be whole-body cryotherapy. Although having similarities in terms of naming and pursued aims, these two approaches are fundamentally different. The present article reviews the available literature on the main safety concerns associated with the use of true whole-body cryotherapy. English- and French-language reports of empirical studies including case reports, case series, and randomized controlled trials (RCTs) were identified through searches of PubMed, Scopus, Cochrane, and Web of Science electronic databases. Five case reports and two RCTs were included for a total of 16 documented adverse events (AEs). A critical in-depth evaluation of these AEs (type, severity, context of onset, participant’s medical background, follow-up) is proposed and used to illustrate that WBC-related safety risks are within acceptable limits and can be proactively prevented by adhering to existing recommendations, contraindications, and commonsense guidelines.

**Supplementary Information:**

The online version contains supplementary material available at 10.1186/s40001-023-01385-z.

## Introduction

Cryotherapy or cryostimulation is a short exposition to extremely low temperatures. The term “cryostimulation” was recently coined to refer to the use of cold exposure among healthy participants (e.g., athletes), whereas the term “cryotherapy” is restricted to the therapeutic use of cold in the management of injuries, disorders, of painful conditions.

Two categories of cryotherapy/cryostimulation devices should be clearly distinguished from one another: (1) Whole-Body Cryotherapy (WBC), delivered inside entire-body cryogenic chambers called “cryochambers” where air temperature is lowered to − 50° to – 150 ℃, and (2) Partial-Body Cryotherapy (PBC), delivered in can-shaped barrel coolers called “cryosaunas” filled with a mixture of air and liquid nitrogen mist at about − 190 ℃.

The differences between WBC and PBC mainly involve the exclusion of the head in PBC treatment, different ways to create cold (nitrogen vapor directly injected inside the cryosauna for PBC, *versus* refrigerated air injected into the cryochamber usually from an outdoor air-conditioner in the case of WBC), as well as different device sizes and mobility possibilities.

Importantly, WBC and PBC also strongly differ in that the PBC approach simultaneously imposes two different types of stress on participants: cold and hypoxia. Such a combination of constraints may lead to the activation of different cell signaling cascades compared to a cold stimulus alone [1].

The last difference between WBC and PBC relates to the thermal homogeneity within the cryogenic units, with cryosaunas displaying a higher degree of heterogeneity in temperature from the bottom to the top, and from the wall to the center of the cabin.

The effectiveness of WBC has been established in the treatment and rehabilitation of several diseases like multiple sclerosis, arthrosis, chronic back pain, or fibromyalgia [2–5]. We also know that WBC is widely used in sports medicine [6], in case of injury and to recover faster after physical exercise and training [7], and may have a potentially positive effect on affective disorders [8, 9], deterioration of cognitive functions [10], poor sleep quality [11], and metabolic disorders as well [12]. The potential mechanisms of action still remain quite unclear. However, the influence of WBC via alleviation of inflammatory processes and reduction of oxidative stress has been reported [13, 14].

With the increasing number of cryocenters, the number of WBC sessions delivered has multiplied in France and worldwide. According to the French Society of Whole-Body Cryotherapy, the number of cryotherapy sessions delivered in France was estimated to be over one million for the year 2019 (approximately 200 cryocenters nationwide, with an average of 5000 sessions per year in each center), 40% of them being WBC sessions. In Poland, where whole-body cryotherapy is covered under the national health fund, the number of reimbursed WBC sessions was estimated to be around 650,000 sessions per year over the last decade. The high number of exposures may result in an increased number of complications despite taking precautions (window and/or camera allowing continuous monitoring over the treatment time, cold protection equipment, security door for fast exit).

Surprisingly, only a few cases of complications have been reported in the literature to date. Part of the reason for this are the precautionary measures taken over the last two decades for safety and security (see list of absolute contraindications jointly released and implemented by the Bad Voslau consensus (Additional file 1: 15, Appendix 2) and the International Institute of Refrigeration [16]). However, the lack of evidence on adverse events is thought to be underestimated due to either underreporting or a lack of uniform reporting standards. The present consortium emerged in response to calls by several experts for further investigations on WBC safety [17], and our aim here was to critically analyze each reported case of WBC-induced adverse events using well-established reporting standards.

To do so, the Common Terminology Criteria for Adverse Events (CTCAE) grading system [18] was used for assessing the seriousness of any adverse event, which allowed to distinguish between « minor» (Grade 1 or Grade 2) and « serious» (Grade 3, Grade 4, or Grade 5) adverse events in the present review. As can be seen in Additional file 1: Appendix 1, adverse events classified into the Grade 3 (or more) category define severe complications requiring at least hospitalization or invasive healing therapy, while Grade 1 and Grade 2 adverse events require no or non-invasive interventional procedures. As the weight of scientific evidence for a causal relationship in each of the cases to be presented differs somewhat, a judgment has been made at the end of each section. This assigned associations to one of four categories: (1) convincing evidence for a causal relationship, (2) probable evidence, (3) possible evidence, and (4) insufficient evidence. « Convincing» and « probable» evidence for a causal disease/exposure relationship should result in policy recommendations, while « possible» and « insufficient» evidence indicate the need for more research.

## Methods

The literature search was performed in the PubMed, Scopus, Cochrane, and Web of Science electronic databases using the following keywords and Boolean operators “adverse OR side OR negative AND effects OR complications AND whole-body cryotherapy OR extreme cold exposure OR cryostimulation”. These keywords and operators were used in an identical manner to query the four databases listed above.

An iterative approach—the “scoping review” approach—was preferred over the more conventional systematic review approach to identify the published studies that reported adverse events in participants who received WBC sessions and to qualitatively analyze the nature of adverse events, as well as their context of occurrence and evolution over time. Scoping reviews are an ideal tool to determine the volume and types of available evidence in emerging topics [19].

**Fig. 1:**
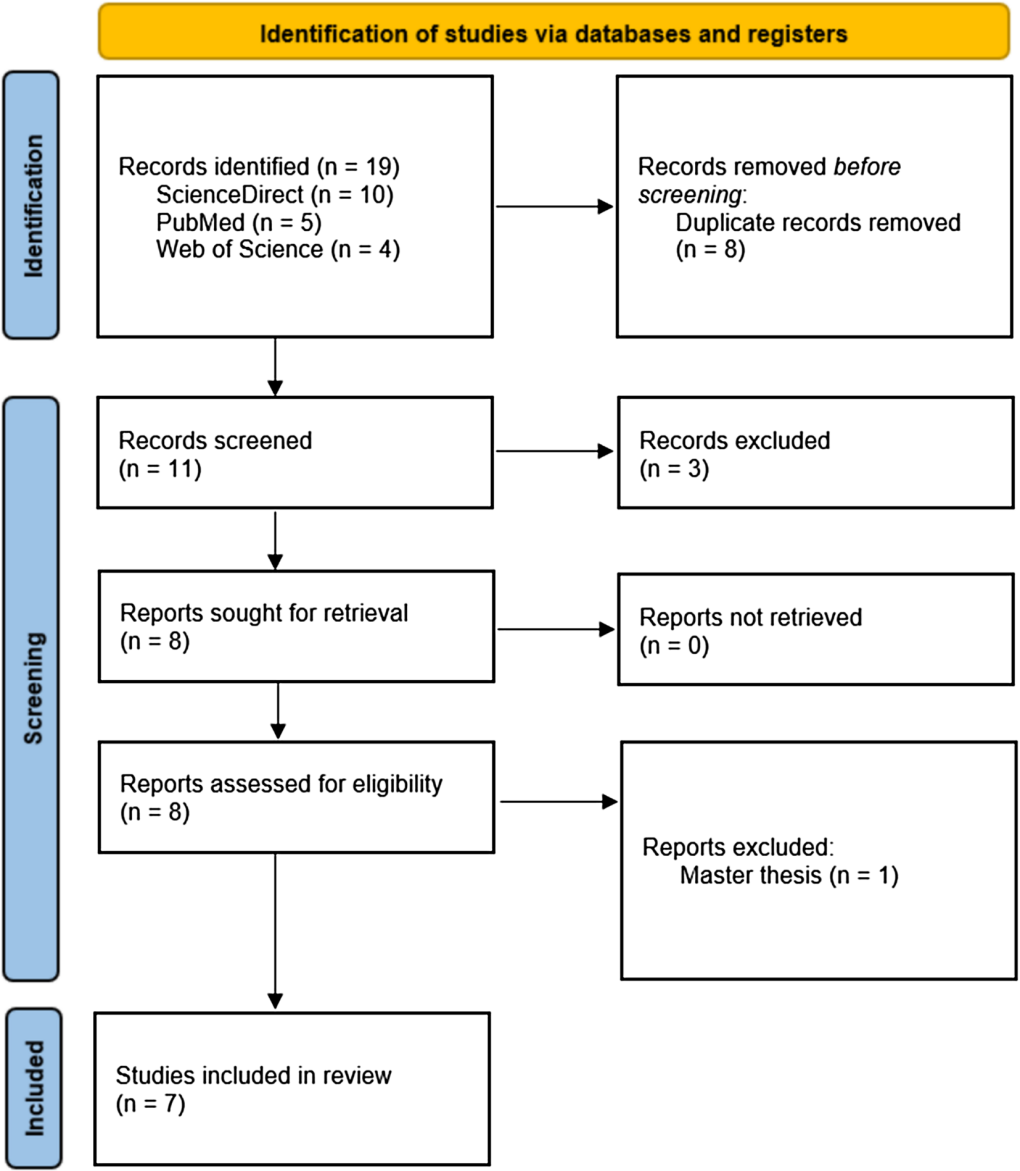
PRISMA flowchart

Studies published in languages other than English and French were excluded. No temporal restriction was performed. A combined search for these keywords returned 19 records, published up to February 2023. In the second step, literature screening was done based on a set of inclusion and exclusion criteria to identify the papers to be read in detail. Eight papers were found to be duplicates, and another 3 records were excluded as they used cryosauna interventions (despite having indicated whole-body cryotherapy in the title). One master’s thesis was also excluded. In total, 7 articles were reviewed. Six were written in English and 1 in French. The corresponding Prisma flowchart is shown in Fig. 1.

Two reviewers (G. P. and F. D. L.) independently examined the abstract and full-text of identified studies. Disagreements in interpretation and/or grading were resolved by consensus-building discussions.

## Results

Our search strategy located 7 articles reporting a total of 16 documented adverse events (Table 1). Five of these 7 articles were case reports studies [20–24], and the remaining 2 papers were randomized controlled trials (RCT) which mentioned safety data in their results section [25, 26]. Reported cases were mostly middle-aged to aged individuals treated for benign, self-limiting conditions such as back pain, joint pain (rheumatism/arthritis), or sleep disturbances. There was no clear over-representation of one sex over another. In most reported cases, patients fully recovered within less than 1 month.

**Table 1:** Retrospective case series of adverse events during or following WBC

First author (year)	Number of cases	Preexisting risk factor	Latency of AE onset	Nature of AE	Gravity of AE (grade)	Recovery time	Evidence for causality
Greenwald [22]	1	Neutropenia	A few days	Cold panniculitis	Minor (grade 2)	Several weeks	Possible
Carrard [20]	1	Work-related stress	Right after WBC	Global amnesia	Serious (grade 3)	24 h	Possible
Cronier [23]	1	Ocular migraine	Right after WBC	Cerebral bleeding	Serious (grade 4)	3 months	Probable
Chen [24]	1	None	Right after WBC	Moyamoya synd	Serious (grade 3)	1 month	Probable
Camara [21]	1	Hypertension	One week	Aortic dissection	Serious (grade 4)	2–3 months	Probable
Happe [25]	2	NR	During study	Headache	Minor (grade 1)	NR	Convincing
Hirvonen [26]	4	NR	During study	Discomfort/dizz	Minor (grade 1)	NR	Convincing
Hirvonen [26]	1	NR	During study	Hypertension	Minor (grade 1)	NR	Convincing
Hirvonen [26]	2	NR	During study	Lasting shivering	Minor (grade 1)	NR	Convincing
Hirvonen [26]	1	NR	During study	Headache	Minor (grade 1)	NR	Convincing
Hirvonen [26]	1	NR	During study	Urticaria	Minor (grade 1)	NR	Convincing

*AE* adverse events, *NR* not reported, *moyamoya synd.* moyamoya syndrome, *discomfort/dizz.* discomfort/dizzines

### Cutaneous lesions

#### Cold panniculitis

Greenwald et al. [22] reported a case of cold panniculitis in a 47-year-old man who received 8 WBC sessions over the 2 weeks preceding symptoms onset. Panniculitis is an inflammation of the fat beneath the outer layer of the skin. It manifests through an erythematous eruption (indurated plaques, nodules, and papules). Rashes of panniculitis either result from irritation (causing a bacterial infection), abnormal activity of pancreatic enzymes, self-injection of different substances (e.g., heroin, crack), drug-related side effects, or inflammation of subcutaneous fat after exposure to cold temperatures [27]. It can usually be treated with over-the-counter medications (anti-inflammatory pain killers) and simple home treatments like leaving affected areas to open air for faster healing. Of course, when panniculitis is caused by a medication or by cold exposure, stopping the medication or discontinuing cold exposure is required. After a diagnosis of panniculitis was made for this patient, cessation of cold exposure was the only treatment received. The patient’s condition improved spontaneously within a couple of weeks. On the basis of this case description, Greenwald et al. concluded that reported symptoms were most consistent with cold panniculitis. However, an overlooked information is that this patient had a history of autoimmune neutropenia ([22], p. 244), which is a disease known to increase the risk for cutaneous bacterial infections [28]. Therefore, what can be concluded is only « possible evidence» for a causal relationship between WBC and panniculitis.

#### Urticaria

Hirvonen et al. [26] published a RCT in which 40 patients with rheumatoid arthritis received 3 daily 3 min long sessions of whole-body cryotherapy at − 60 ℃ (*n* = 20) or − 110 ℃ (*n* = 20) over 1 week. Thirty-five patients (i.e., 87.5%) were females, and the mean age was 51 years. One patient in the WBC at − 110 °C group reported urticaria. No further information is given on how and when it appeared, whether it was medically confirmed, and what treatments were prescribed (if any). As a general and indiscriminate account of adverse events in their RCT, Hirvonen et al. concluded that no serious or permanent adverse events occurred and that all except one participant felt WBC acceptable or tolerable ([26], p. 299). Urticaria is a common condition with a lifetime prevalence of approximately 15% and females being more affected than males [29]. Many trigger factors have been identified in medical research such as specific food proteins, pollen, or drugs [29]. Urticaria can also result from specific medical conditions, hormonal imbalances, or physical stress (heat, cold, pressure, sunlight) [29]. Even the mere application of hydrating creams, oils, or self-tanning products—which is common practice in many non-medical cryocenters—can potentially cause urticaria. Finally, there is evidence that the risk for cold urticaria is increased in patients with cold-dependent antibodies such as cryoglobulins or cold agglutinins [30]. That being said, in the absence of medical information for this patient, and given that no other reason for urticaria was reported in this study, it seems rather logical to conclude that there is convincing evidence for a causal relationship between WBC participation and urticaria.

### Vascular and neurological complications

#### Transient global amnesia

In a Swiss hospital, a 63-year-old male presented with transient global amnesia after undertaking a WBC session [20]. The patient did not have any other symptoms. This WBC session was the second one in his life, and the first went without any problems. The amnesia covered a period of time starting 30 min before the WBC session and ending 3 h after it. Brain magnetic resonance angiography revealed no sign of ischemic or hemorrhagic lesions and no stenosis of the arteries perfusing the brain. Blood analyses showed a somewhat elevated glycemia which persisted on the following day, vitamin D and B_12_ deficits, and dyslipidemia. In addition, an ECG and transthoracic echocardiogram were performed, revealing a normal cardiac rhythm pattern and heart morphology. Overall, the patient received intensive hospital-based monitoring for 24 h, but no specific treatment. The patient completely recovered in 24 h. In their conclusion, the authors assert that WBC can potentially result in transient global amnesia, as cold is a risk factor for this. Though it is true that cold exposure (more broadly, intense temperature changes) can trigger transient amnesia attacks, this is also true for several other precipitating events such as vigorous exercise (including sexual intercourse), or stressful events [31]. Interestingly, by his own admission, the patient was experiencing a lot of stress at work at the time his amnesia occurred [20]. In addition, there is growing concern over proton-pump inhibitors (PPIs) neurological side effects, including memory impairments [32]. It happens that the patient in this case study was on Omeprazole (a medication in the category of PPIs) for 2 years at the time the event was recorded [20]. Consequently, although cold exposure may have induced amnesia, two other potential reasons for this adverse event have been missed by Carrard et al. In conclusion, all that can be said from this report is that WBC had a « possible» causal relation to the patient’s transient amnesia.

#### Intracerebral hemorrhage

Intracerebral hemorrhage is sudden bleeding into the tissues of the brain or into its ventricles. Symptoms—which typically get worse over time—can include headache, one-sided weakness, vomiting, seizures, neck stiffness, and decreased level of consciousness [33]. Undoubtedly, it can be considered as a life-threatening complication since 44% of those affected usually die within a month [33]. The first case of intracerebral hemorrhage during a WBC session was recently reported by a French group [23]. It was observed in a 61-year-old woman during her first WBC session, as she moved from the pre-chamber (− 60 °C) to the main chamber (− 110 °C). The subject suddenly complained of severe headache and nausea, accompanied by sensory and motor disturbances (left-sided hemiparesis). The next day, the MRI images revealed a 36 × 22 × 10 mm-sized edema in the right superior frontoparietal region. Complementary brain activity measures and cerebrospinal fluid analyses revealed no other lesions or signs of disease. The subject was treated with nimodipine (180 mg per day) and regained the use of her left leg and arm in a few weeks (no more precise information was given). The patient was able to get back to work after 3 months of convalescence. Considering that no alternative explanation could be found for this subject’s intracerebral hemorrhage, the authors proposed that WBC was the precipitating cause. Nevertheless, it should be pointed out that this woman had a long history of ocular migraine at the time she engaged in her first WBC session ([23], p. 844), a condition which has been found to increase the risk of hemorrhagic stroke by 50% in the meta-analysis by Sacco et al. [34]. Indisputably, it would be unwise to dismiss the role of WBC (more particularly of the hypertensive pike resulting from extreme cold exposure) in relation to the onset of cerebral bleeding in this patient. However, this reported WBC-related complication is at least partly attributable to the well-established underlying vascular vulnerability of individuals prone to migraine.

#### Moyamoya angiopathy

Moyamoya syndrome is a progressive stenosis of the intracranial carotid arteries. It can result in decreased cerebral blood flow, with resulting symptoms similar to ischemic stroke. A study by Chen et al. from USA has recently described a manifestation of moyamoya syndrome following WBC at − 90 °C [24]. It was observed in a 32-year-old woman with no past medical history. As she was receiving her first-ever WBC session, she felt unwell after 4 min and was unable to continue the session. The main reported symptoms were inability to produce words, and a sudden decrease of strength in her right arm. After immediate admission to hospital, a computed tomography (CT) angiogram of the head and neck showed a near-occlusion of the left internal carotid artery as well as a severe narrowing of the left posterior cerebral artery. Her clinical condition rapidly improved in the emergency room. She was discharged because of spontaneous improvement and received no treatment except aspirin (325 mg). At 1 month follow-up, the patient showed no sign of aphasia, weakness, or other neurological deficits on examination. The authors concluded that WBC may induce cerebral vasoconstriction, and suggested that cold-induced hyperventilation could trigger this unfavorable side effect. In line with this conclusion, we assume that there is probable evidence for a cause-and-effect association between cold exposure and the onset of symptoms in this patient. That being said, the reported session length seems unusually long (> 4 min) compared to the recommendations from the Bad Voslau consensus (Additional file 1: 15, Appendix 2). This is especially questionable in the context of the given facts and circumstances (first-ever WBC session, healthy but non-athlete woman). Therefore, we believe that the risk of angiopathy associated with participation in WBC sessions is actually minimal provided that applicable recommendations are followed.

#### Abdominal aortic dissection

In Mexico, Camara-Lemarroy and colleagues published a case report about a 56-year-old male admitted to an intensive care unit after experiencing sudden abdominal pain accompanied by dyspnea and lightheadedness [21]. This occurred 1 week after he had completed a 15-session long WBC program (3 min per session, − 150 to − 160 °C). The chest CT angiogram revealed no evidence of any abnormalities of the major arteries. All laboratory measures were within the health-related reference interval. The patient was discharged 2 days later with no abdominal pain. However, 9 days later, he was admitted again, with identical abdominal pain. The abdominal CT angiogram showed an anterior aortic dissection of 36 mm that ended 13 mm before the iliac bifurcation. The next day, an endovascular prosthesis was placed, with no complications. During the 2 following months, the patient still experienced episodes of abdominal pain, most of which were associated with cold temperature exposure. After receiving clonidine (0.5 mg per day) and avoiding cold temperatures, the patient improved greatly. Based on these elements, the authors suggested that the implication of WBC in the occurrence of aortic dissection was at least probable. Yet two comments should be made here to maintain a due sense of moderation. First, the patient described in this case report had a documented history of hypertension and hypercholesterolemia that were being treated with medication. Arterial hypertension is one of the 14 absolute contraindications to WBC defined by the Bad Voslau consortium (Additional file 1: 15, Appendix 2). Second, the subject described himself to be an “avid runner” at the time the event occurred ([21], p.e67). Excessive exercise has been identified as one of the most important risk factors for abdominal aortic aneurysms [35].

#### Other complications (dizziness/malaise, hypertension, headache, long-lasting shivering)

All other WBC-induced adverse events reported in the literature (*n* = 10) were of mild severity. They were not described in detail on a case-by-case basis and were simply mentioned in the Results section from the published manuscripts by Happe et al. [25] and Hirvonen et al. [26]. In total, there were 3 cases of cold-induced headache, 4 cases of discomfort/dizziness, 1 case of reactive hypertension, and 2 cases of long-lasting shivering. None of these events resulted in the discontinuation of the study protocol; and none required medical investigation, surveillance, or treatment.

Table 1 presents a synthesis of the characteristics of the studies included in our systematic review and also separately shows a qualitative assessment of evidence for causality.

## Discussion and conclusions

Whole-body cryotherapy (WBC) involves short exposures of the entire body (including the head) to very cold and dry air (temperatures below − 50 °C) in specially adapted cryochambers.

Several institutions have expressed concerns regarding WBC safety over the last decade [36, 37]. However, their analysis and conclusions are flawed in one critical way: partial-body (body in a cryosauna but head remaining outside, direct injection of liquid nitrogen mist inside the cabin) and whole-body (full exposure in a cryochamber filled with breathable air) technologies have been amalgamated under the alleged banner « whole-body cryotherapy». As an illustration of this point, Ben Kheder et al.’s analysis of WBC safety [37] is based on 4 studies [20–22, 38], one of which used a cryosauna cooling equipment [38]. Another example is the case study by O’Connor and colleagues [39] regularly cited as scientific evidence in support of the dangerousness of WBC. It actually describes a cold burn injury in a 71-year-old man who accidentally had his back burned by liquid nitrogen while standing in a cryosauna (i.e., partial-body cryotherapy). Indisputably, special care must be taken when using cryosauna (PBC) equipment as there are inherently high risks of cold burns and anoxia due to the direct injection of vaporized nitrogen gas. On the contrary, the data discussed in the present review suggest that true WBC is associated with relatively infrequent, and mostly minor and transient adverse effects. Evidence of its adverse effects is strongest for vascular/neurological complications: intracerebral hemorrhage [23], moyamoya angiopathy [24], and abdominal aortic dissection [21]. However, careful examination of each of these reported serious adverse events allowed to notice significant patient’s related risk factors [21, 23], or inappropriate WBC exposure parameters in terms of session length [24] or ambient temperature [21].

In conclusion, looking back on the past four decades (Japanese professor Toshio Yamauchi designed the first cryosauna in 1979, and WBC cryogenic chambers were first developed shortly after the turn of the 1980s in Europe) adverse events appear to be rare in relation to the extent to which WBC has grown worldwide. Moreover, the level of scientific evidence of serious adverse events related to the use of WBC is still low to date, limited to published case reports. Our opinion is that some of the adverse reactions reported here could have been prevented with a better understanding or better application of the Bad Voslau’s list of contraindications. We reaffirm the importance of considering medical contraindications before involving any subject in a WBC program.

In this regard, we propose to upgrade the Bad Voslau’s list by including migraine due to the documented association with increased risk of hemorrhagic stroke. Also, a special concern regarding lipid disorders should be raised, as one case of abdominal aortic aneuryms has been observed. Paradoxically, extreme cold exposure may help obese and overweight individuals to lose weight and improve circulating lipid profile [40].

### Supplementary Information

**Additional file 1: A1.** Grades to classify the severity of harms (Common Terminology Criteria for Adverse Events). **A2.** Bad Voslau absolute contraindications to WBC.

## Data Availability

Retrieved studies used for this scoping review are available upon reasonable request and will be made available to researchers who provide a sound proposal. Proposals should be directed to fabien.legrand@univ-reims.fr (corresponding author).
