# Correction: Evaluating safety risks of whole-body cryotherapy/cryostimulation (WBC): a scoping review from an international consortium

**DOI:** 10.1186/s40001-024-01725-7

**Published:** 2024-03-12

**Authors:** Fabien D. Legrand, Benoit Dugue, Joe Costello, Chris Bleakley, Elzbieta Miller, James R. Broatch, Guillaume Polidori, Anna Lubkowska, Julien Louis, Giovanni Lombardi, Francois Bieuzen, Paolo Capodaglio

**Affiliations:** 1 Laboratoire C2S, EA 6291, Universite de Reims Champagne Ardennes, 51100 Reims, France; 2 Laboratoire Mobilite VieillissementExercice (MOVE), UR 20296, Faculte des Sciences du Sport, Universite de Poitiers, 86000 Poitiers, France; 3 Extreme Environments Laboratory, School of Sport, Health and Exercise Science, University of Portsmouth, Portsmouth, England, UK; 4 Faculty of Life and Health Sciences, Ulster University, York St, Belfast, BT15 1ED UK; 5 Department of Neurological Rehabilitation, Medical University of Lodz, Milionowa 14, Lodz, Poland; 6 Institute for Health and Sport (IHES), Victoria University, Melbourne, Australia; 7 MATIM, Universite de Reims Champagne Ardennes, 51100 Reims, France; 8 Department of Functional Diagnostics and Physical Medicine, Pomeranian Medical University in Szczecin, Żołnierska 54, 71‑210 Szczecin, Poland; 9 Research Institute for Sport and Exercise Sciences (RISES), Liverpool John Moores University, Liverpool, L3 3AF UK; 10 Laboratory of Experimental Biochemistry & Molecular Biology, IRCCS Istituto Ortopedico Galeazzi, Via Cristina Belgioioso 173, 20157 Milano, Italia; 11 Service des Sciences du Sport, Institut National du Sport du Quebec, Montreal, QC Canada; 12 Research Laboratory in Biomechanics, Rehabilitation and Ergonomics, Istituto Auxologico Italiano IRCCS, Piancavallo, VB Italy; 13 Department of Athletics, Strength and Conditioning, Poznań University of Physical Education, Królowej Jadwigi 27/39, 61-871 Poznań, Poland; 14 Physical and Rehabilitation Medicine, Dept. Surgical Sciences, University of Torino, Turin, Italy

Correction: European Journal of Medical Research (2023) 28:387 10.1186/s40001-023-01385-z

In the original publication of the article [1], the affiliation details of the authors, Giovanni Lombardi and Paolo Capodaglio were incorrectly given as “Laboratory of Experimental Biochemistry, IRCCS Istituto Ortopedico Galeazzi, 20157 Milan, Italy” and “Laboratorio di Ricerca in Biomeccanica, Riabilitazioneed Ergonomia, Universita di Torino, Torino, Italy” respectively. The corrected affiliations of the author were given in this Correction.
