# Supplementary material for: Evaluating safety risks of whole-body cryotherapy/cryostimulation (WBC): a scoping review from an international consortium
Source: Eur J Med Res. 2023 Sep 28;28:387. doi: 10.1186/s40001-023-01385-z (PMC10537204; doi:10.1186/s40001-023-01385-z)
Supplement: Supplementary file 1 — Additional file 1: A1. Grades to classify the severity of harms (Common Terminology Criteria for Adverse Events). A2. Bad Voslau absolute contraindications to WBC. [file 40001_2023_1385_MOESM1_ESM.docx]

**Appendix**

**A1 - Grades to classify the severity of harms (Common Terminology Criteria for Adverse Events)**

Grade 1 –“Mild”: Asymptomatic or minor symptoms; clinical or diagnostic observations only; no intervention needed

Grade 2 –“Moderate”: Minimal, local, or non-invasive intervention indicated

Grade 3 –“Severe”: Medically significant but not immediately life-threatening; hospitalization or prolongation of hospitalization indicated; disabling

Grade 4 –Life-threatening consequences (i.e., immediate risk of death); urgent intervention indicated

Grade 5 –Death related to adverse event

**A2 - Bad Voslau absolute contraindications to WBC**

1. Untreated high blood pressure

2. Heart attack within the past six months

3. Decompensated diseases of the cardiovascular and respiratory system

4. Unstable angina

5. Pacemaker

6. Peripheral artery occlusive disease (Fontaine stages III and IV)

7. History of deep-vein thrombosis

8. Acute febrile diseases of the respiratory tract

9. Acute renal and urinary disorders

10. Severe anemia

11. Signs or symptoms of cold allergy

12. Severe wasting diseases

13. Seizure disorders

14. Large-area bacterial and viral skin infections, wound-healing problems

15. Alcohol and drug influence
